# Fijian medicinal plants and their role in the prevention of Type 2 diabetes mellitus

**DOI:** 10.1042/BSR20220461

**Published:** 2022-11-16

**Authors:** Pritika Mala, Gausal A. Khan, Romila Gopalan, Desta Gedefaw, Katy Soapi

**Affiliations:** 1 Department of Pharmacy and Pharmacology, College of Medicine, Nursing and Health Sciences, Fiji National University, Suva, Fiji Islands; 2 School of Agriculture, Geography, Environment, Ocean and Natural Sciences, The University of the South Pacific, Laucala Campus, Suva, Fiji Islands; 3 Department of Physiology and Physiotherapy, College of Medicine, Nursing and Health Sciences, Fiji National University, Suva, Fiji Islands; 4 Department of Clinical Nutrition, College of Applied Medical Sciences, King Faisal University, Al Hofuf, Al Ahsa, Saudi Arabia; 5 Catholic Regional College Melton, Melbourne Australia; 6 Pacific Community Centre for Ocean Science, Pacific Community, Suva, Fiji Islands

**Keywords:** alpha amylase, free radicals, medicinal plants, type 2 diabetes

## Abstract

Medicinal plants (MPs) are natural sources of active compounds with potential therapeutic benefits in alleviating various illnesses for decades. Fijian people also are using these MPs for the management/prevention of Type 2 diabetes mellitus (T2DM) and associated complications. However, till date, none of these Fijian MP’s antidiabetic potential have been explored or evaluated. Here, we investigated the antidiabetic potential of Fijian MPs scientifically. Phytochemicals such as polyphenols were detected to inhibit the activity of α-amylase and α-glucosidase, the two key carbohydrate enzymes linked to T2DM. Therefore, in the present study, the total phenolic content (TPC), α-amylase and α-glucosidase inhibitory activity of five Fijian MPs: Vobo (*Mussaenda raiateensis*, MR), Vula walu (*Blechnum orientale*, BO), Gasau (*Miscanthus floridulus*, MF), Molikaro (*Citrus limon*, CL) and Beki ni sina *(Dicranopteris caudate*, DC) collected from mainland region of Vitilevu, Fiji Islands, were evaluated *in vitro*. The hydromethanolic (ME) and dichloromethane (DM) extracts of these selected MPs were investigated. The ME extracts of BO (0.102 ± 0.009 mM CE) and DC (0.098 ± 0.09 mM Catechin Equivalence [CE]) showed a higher TPC compared with the control [vanillic acid (0.052 ± 0.003 mM CE, *P value < 0.05)]. However, the TPC of MF, MR and CL were found in the range of 0.020 ± 0.009 to 0.009 ± 0.01 mM CE. The ME extracts of MF and MR inhibited α-glucosidase significantly in comparison with acarbose as evidenced from the IC_50_ values (IC_50_ of MF = 1.58 ± 0.03 ng/µl; IC_50_ of MR = 1.87 ± 0.43 ng/µl and IC_50_ of acarbose = 3.34 ± 0.15 ng/µl). Moreover, DM extracts of MR (IC_50_ = 1.31 ± 0.29 ng/µl) also showed significantly higher α-glucosidase inhibitory activity. In contrary, MR (IC_50_ = 16.18 ± 0.16 ng/µl) and CL (IC_50_ = 9.21 ± 0.51 ng/µl) also showed significant α-amylase inhibitory activity in ME and DM extracts, respectively. These, results suggest that Fijian MPs could be a potential source of natural inhibitors of enzymes involved in carbohydrate digestion and thus may possibly be used in managing T2DM.

## Introduction

Diabetes mellitus (DM), characterized by hyperglycemia, is the most common endocrine disorder, affecting millions of people worldwide [1]. Hyperglycemia is either caused by an absolute insulin hormone deficiency in the system, Type 1 diabetes mellitus (T1DM) or due to the systemic resistance to the insulin hormone and pancreatic β-cell dysfunction, Type 2 diabetes mellitus (T2DM) [2]. According to International Diabetes Federation, 451 million people were living with DM (prevalence rate at 8.4%) in the year 2017, and this figure is expected to rise to 693 million by the year 2045 with projected prevalence rate at 9.9% [3]. These increased numbers of diabetic patients are mainly due to T2DM (85–91%), which although preventable, remains a leading cause of morbidity and mortality worldwide in addition to being a major economic burden [3]. T2DM prevalence trends in Fiji indicates an increase from 7.7% to 17.7% between 1980 and 2022, with the projection to 48% to 629 million by 2020 [4]. The country’s T2DM associated complications resulted in 938 amputations from 2010 to 2012 period of which, 15.9% were undiagnosed cases [5]. Medical reports have also revealed various other complications such as retinopathy [6,7], foot sepsis [8], cardiovascular complications and renal failure [9,10]. These all confirm the severity of the disease in the small island country. With population growth, urbanization, increasing prevalence of obesity and physical inactivity, control of T2DM complications is expected to increase which will incur high medical care costs and a reduced quality of life in Fiji [5,10]. Postprandial hyperglycemia (PPHG), a condition in which sugar level remains high for a longer period after consumption of meal, have been found to play vital role in the onset and development of T2DM complications [11,12]. One of the therapeutic strategies for managing PPHG involves inhibition of carbohydrate hydrolysing enzymes, such as α-amylase (largely produced by pancreas) and α-glucosidase (produced by small intestine lining) [13]. Synthetic hypoglycemic drugs such as acarbose, miglitol and voglibose have strong inhibitory action against these enzymes. However, these drugs have side effects mainly gastrointestinal and abdominal discomfort, apart from it being expensive and not readily available, particularly in developing countries, like Fiji [14].

Thus, the search for natural product inhibitors that are readily available and potentially safer is the ideal choice. The earliest record of MPs to manage T2DM is more than 3,500 years [15] and is still considered as an alternative treatment of diabetes, due to its various benefits such as its accessibility, high efficacy, fewer side effects, low cost, and also it’s an excellent aspirant for oral therapy [16,17]. Numerous studies based on animal model, clinical trials and reviews have reported that phytochemicals such as polyphenols inhibit the two carbohydrate hydrolysing enzymes and boost antioxidant (AO) system, thus managing PPHG and associated complications in the body [18–20]. Phenolic compounds from plants such as curcumin, epicatechin, hesperetin, quercetin, resveratrol, catechin, kaempferol, vanillic acid and 4-hydroxybenzoic acid are reported as significant α-amylase and α-glucosidase inhibitors [21–23].

Furthermore, catechin (a flavanol found in apples, dark chocolate, ginger, cocoa and tea), curcumin (the major polyphenol of turmeric) and resveratrol (a natural phenol found in grapes and peanuts) are also known to reduce diabetic inflammation by scavenging reactive oxygen species (ROS) [24–27]. Similarly, quercetin 3-O-β-D-glucopyranoside showed the highest free radical scavenging activity [22]. Thus, high phenol containing plants has a potential to manage PPHG and can reduce diabetic complications induced by ROS. Wide range of MPs used as traditional medicines (TMs) in Fiji is a much-coveted heritage whose value has been fiercely protected and secretly preserved by family and tribal descendants [28]. Various local traditional medicinal plants (TMPs) used by the traditional herbalists are claimed to be effective antidiabetic therapy, however, to date there has been no scientific study to explore and validate the antidiabetic properties.

Therefore, it is pertinent to explore these TMPs, which can be utilized for lowering blood glucose rendering a cheaper and safer way to manage T2DM thus giving better patient management outcomes. The natural products don’t have any side effect and economically sustainable. So, people will have less economical burden. Hence to fill this gap, our study focused with an objective to investigate the antidiabetic properties of some native plants used by established local herbalists. We hypothesized that local TMPs used by herbalists for managing T2DM; (1) may have proven scientific effect and (2) may contain phenolic compounds that can significantly inhibit the activity of α-amylase and α-glucosidase and hence could help to treat T2DM. Therefore, the present study aimed to assess and compare the total phenolic content (TPC), α-amylase and α-glucosidase inhibitory properties *in vitro* of the hydro-methanolic and dichloromethane extracts prepared from Vobo (*Mussaenda raiateensis*, MR), Vula walu *(Blechnum orientale*, BO), Gasau (*Miscanthus floridulus*, MF), Molikaro (*Citrus limon*, CL) and Beki ni sina *(Dicranopteris caudate*, DC), traditional MPs used by the herbalists in the mainland region of Vitilevu, Fiji.

## Materials and methods

### Chemicals

Solvents were of analytical grade and obtained from Thermofisher (New Zealand). Total phenolic content was determined using Bio vision Phenolic Compounds Assay Kit (Colorimetric); Catalog # K527-200, Milpitas, CA, U.S.A. α-Amylase from *Aspergillus oryzae* (EC: 3.2.1.1; 30 U/mg), α-glucosidase from *Saccharomyces cerevisiae* (≥ 100 U/mg protein) and all other chemicals were bought from Merck Co (Australia). Deionized distilled water was used to prepare specific volumes and concentrations of all the reagents used in the present study.

### Plant materials

All the plant materials used in the present study were collected from the wild habitats from the main island of Vitilevu, Fiji, in the year 2019. Taxonomic identification was made by the botanist at the University of the South Pacific Herbarium. After harvesting a bulk sample, the plants were washed under running water to remove all contaminants. All plant samples were cut into small pieces and air-dried for 4 weeks under the shade at room temperature (RT). The dried plant material (leaves and aerial parts) was grinded into coarse powder texture using an electric blender (Ninja - Professional 1500 Watts) whereas the roots and stems were manually powdered using mortar and pestle. The powdered plant materials were packed in zip lock plastic bags, labeled, and kept in the dark until ready for extraction.

### Preparation of hydro-methanolic extracts (ME)

About 20 g of each plant sample were homogenized in 70% methanol (100 ml) at RT for 24 h. Manual shaking was done after 6 and 18 h of soaking period followed by sonication for 1 h (30°C, 100 P). The mixture was filtered using Whatman filter paper (Cat No. 1441-125) and the filtrate was collected in round bottom flask. The extraction was repeated twice using the marc of the previous extraction. The extracts were combined and concentrated with a rotary evaporator (150 rpm, 30°C) to obtain a viscous liquid. Concentrated extracts were transferred to pre-weighed labeled scintillation vials and dried using SpeedVac Concentrator (Thermo Scientific - Savant SC210A) with the vials loosely capped. The extracts were further dried using freeze drier (Biobase - Type: YL 7124). About 0.05 g of the dried plant extracts were each solubilized in 500 µl of 10% dimethyl sulfoxide (DMSO/H_2_O mixture): methanol (1:1) to give a stock concentration of 100 µg/µl.

### Preparation of dichloromethane extracts (DM)

The methanolic extracts residues were homogenized in 100 ml of dichloromethane (DM) at RT for 24 h. Similar steps as above for preparation of ME extracts were followed thereafter to prepare DM extracts. DM plant extracts were each solubilized in 500 µl acetonitrile: ethyl acetate: methanol (1:1:1) to give a stock concentration of 100 µg/µl.

### Total phenolic content (TPC) by phenolic compounds assay kit (colorimetric)

From the 100 µg/µl stock solution, 0.5 µg/µl plant sample concentration was prepared for TPC analysis. TPC was measured using phenolic compounds assay kit (Catalog # K527, BioVision, Milpitas, CA, U.S.A.) as per manufactures instruction [29]. The absorbance for each well was measured at 480 nm using an ELISA Plate Reader (DR-200Bc: Wavelength Range: 400–800 nm, China). All samples were tested in triplicates. Vanillic acid (VA) was used as the positive control. TPC values were expressed as mM Catechin Equivalents (mM CE), which was calculated using the formula (eqn 1):

(1) $$Sample\;phenolic\;compound\;concentration=\frac{B}{V}\times D$$

where, *B* = amount of diazo chromophore (calculated from the standard curve, in nmol of catechin); *D* is the sample dilution factor; *V* is the volume of sample added to the reaction well.

### *In vitro* α-amylase inhibition assay

α-Amylase inhibitory activity of ME and DM extracts were carried out according to the standard method with minor modifications [30,31]. In an eppendorf tube, reaction mixture containing 20 µl of phosphate buffer (100 mM, pH 6.8), 20 µl of α-amylase (4 U/ml), and 20 µl of varying concentrations (5, 10, 12.5, 25, 50 ng/µl) of plant extracts were pre-incubated at RT for 20 min. Then, 20 µl of 1% soluble starch in 100 mM phosphate buffer was added as a substrate and incubated further at RT for 30 min followed by the addition of 50 µl of the 3,5-dinitrosalicyclic acid color reagent. The reaction mixture was placed in boiling water bath (85–90°C) for 5 min to stop the reaction and then cooled to RT. The absorbance of the resulting mixture was measured at 540 nm using ELISA Plate Reader (DR-200Bc: Wavelength range: 400–800 nm, China) and compared with that of the control, which had 20 µl of phosphate buffer instead of the plant extract. The results were expressed as percentage inhibition, which was calculated using the formula (eqn 2):

(2) $$Inhibitory\;activity\;(\%)\;=\;\left(1-\frac{As}{Ac}\right)\times 100$$

where, *A*_s_ = absorbance in the presence of test substance; *A*_c_ = absorbance of control.

Acarbose was used as positive control and all measurements were performed in triplicate.

### *In vitro* α-glucosidase inhibition assay

The α-glucosidase inhibitory activity of ME and DM extracts were carried out according to the standard method with minor modifications [30,31]. In a 96-well plate, reaction mixture containing 50 µl of phosphate buffer (100 mM, pH 6. 8), 10 µl of α-glucosidase (1 U/ml), and 20 µl of varying concentrations of plant extract was pre-incubated at RT for 15 min. Then, 20 µl of p-nitrophenyl-α-D-glucopyranoside (5 mM) was added as a substrate and incubated further at RT for 20 min. The reaction was stopped by adding 50 µl of Na_2_CO_3_ (0.1 M). The absorbance of the released p-nitrophenol was measured at 405 nm using Eliser Plate Reader (DR-200Bc: wavelength range: 400–800 nm) and compared with that of the control, which had all the components except the plant extract replaced by 20 μl of phosphate buffer. The results were expressed as percentage inhibition, which was calculated using the formula (eqn 3):

(3) $$Inhibitory\;activity\;(\%)\;=\;\left(1-\frac{As}{Ac}\right)\times 100$$

where, *A*_s_ = absorbance in the presence of test substance; *A*_c_ = absorbance of control.

Acarbose was used as positive control and all measurements were performed in triplicate.

### Statistical analysis

The results were expressed as means of three independent experiments ± standard deviations ($\bar{x}=\mathrm{SD}$). Statistical difference among the plants were assessed by one-way analysis of variance (ANOVA) using Graph Pad Prism 8 version 8.4.3 (Graph pad software, Inc., La Jolla, CA, U.S.A.) statistical software, and the individual comparisons were obtained by Turkey’s multiple comparisons test. Statistical significance was indicated by *P value < 0.05. Correlation tests were done using Pearson correlation coefficient, −1 ≤   ≤ 1.

## Results and discussion

Herbal preparation are mostly done in water via method of concoction and decoction [32]. Additionally, methanol is documented as a ‘all purpose’ solvent, which dissolves bulk of the secondary metabolites and also increases their release from the plant cellular cell surface [33]. The traditional medicinal preparation documented for the present study used water as the extraction solvent. This suggests that traditional medicinal preparations would have polar components (water soluble and nonvolatile constituents). However, there is a possibility that lower polarity antidiabetic component may also be present in plant extracts. Since the herbalist were all using water, low polarity components may not have been optimally extracted. Therefore, to ensure that useful phytochemicals with both higher and lower polarity were captured, the present study used hydro-methanol-70% (ME) and dichloromethane (DM) extracts.

Moreover, shade dried plants were used for the present study. This is mainly to remove moisture from the samples for an increased shelf life of the plant samples. The dried plants were grinded to increase the surface area with the extraction solvents for a better extraction yield. Repeated maceration was done to increase the yield.

There was a higher yield of phytochemical extracted from the MPs with ME solvent (5.89–19.52%) when compared with the yield of DM (0.46–2.72%) as shown in Table 1 (*P*=0.6648). ME extract of DC (DCME) and DM extract of MF (MFDM) recorded the highest % crude yield. The difference in the % yields between ME and DM extracts could mainly be due to presence of high number of polar components compared with low polarity components in the plants chosen for the present study. Compounds such as proteins and carbohydrates that have higher solubility in water and methanol may have been extracted. The extraction yield in the present study is consistent with the literature, where for example, *Limnophila aromatic* gave a 75% extraction yield in aqueous methanol but low in DM [34,35].

**Table 1:** Percentage crude extraction yield of hydro-methanol-70% (ME) and dichloromethane (DM) plant extracts

Scientific names	Common names	% yield of the extract	
		ME	DM
1. Mussaenda raiateensis (MR)	Vobo	6.65	0.62
2. *Blechnum orientale* (BO)	Vula walu	12.32	0.62
3. *Miscanthus floridulus* (MF)	Gasau	8.53	2.07
4. *Citrus limon* (CL)	Molikaro	5.89	0.46
5. *Dicranopteris caudata* (DC)	Beki ni sina	19.52	2.72

Formula used to do the yield calculation: % Crude Yield = (mass of plant extract/mass if dried plant used) × 100, where; Total mass of each dried plant = 20 g.

### Total phenolic content (TPC)

**Figure 1:**
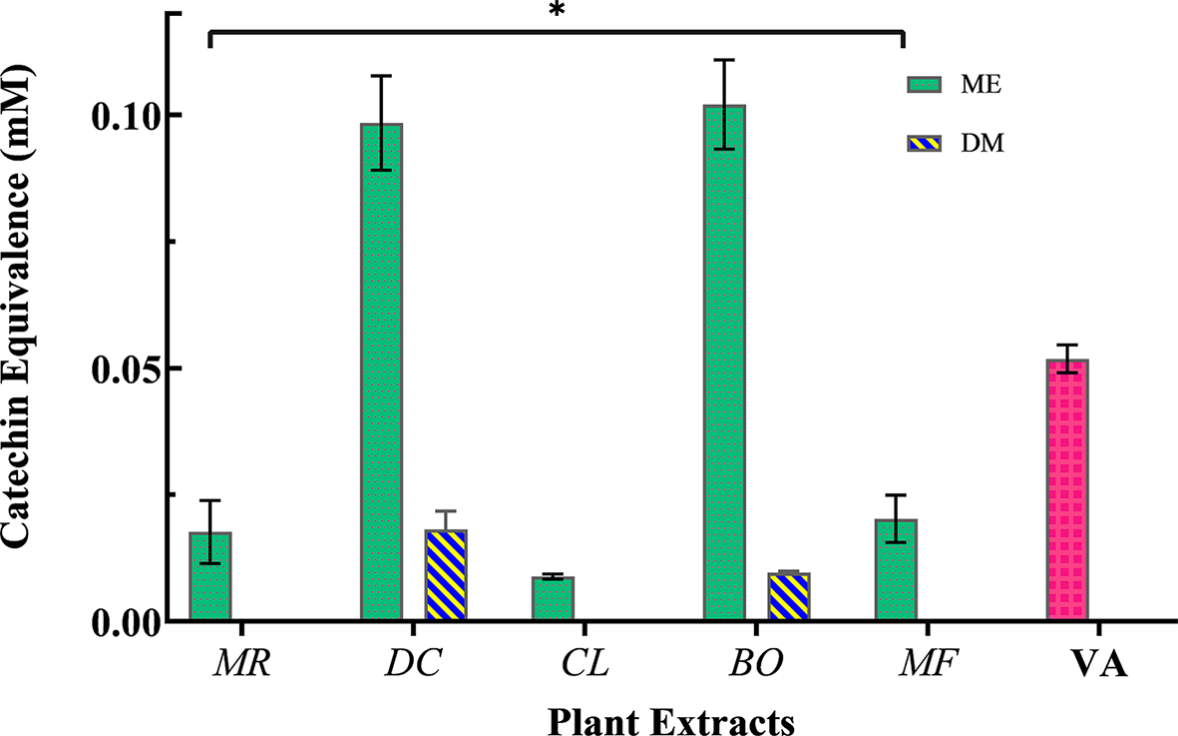
Total phenolic content (TPC) of *Blechnum orientale* (BO), *Dicranopteris caudata* (DC), *Miscanthus floridulus* (MF), *Mussaenda raiateensis* (MR) and *Citrus limon* (CL) in ME and DM plant extracts TPC is expressed as nmol catechin equivalence/50 µl/10 min (since total plant extract of 50 µl was taken) ≡ nmol Catechin Equivalence/µl ≡ mM Catechin Equivalents (mM CE). Statistical difference among the plants were assessed by one-way analysis of variance (ANOVA) and the individual comparisons were obtained by Turkey's multiple comparisons test. Statistical significance was tested at a *P value < 0.05, for all cases in comparison with the vanillic acid (VA), the positive control.

PPHG is the primary factor to maintain in the management of T2DM. Phenolics apart from maintaining PPHG has also shown to be powerful antioxidants (AOs) [18–20,36]. In the present study, BOME (0.102 ± 0.009 mM CE) and DCME (0.098 ± 0.09 mM CE) showed a high TPC compared with the control, VA (0.052 ± 0.003 mM CE, as shown in Figure 1. The values were approximately five times less in the DM extracts. Our finding suggest that most of the phenolic compounds were extracted in polar solvents and this is consistent with studies reported using different plant species [35,37].

### The role of phenolics in the management of T2DM

The prevention of oxidative stress (OS) from elevated production of ROS with the consumption of AOs is one of the main pathological mechanisms to lower the risk of organ damage in T2DM patients [38–40]. Studies have linked free radicals to the development of diabetic complications such as retinopathy, neuropathy, cardiomyopathy and nephropathy [41–43]. The AO potential of medicinal plants can be estimated from the analysis of its TPC [44,45]. Phenolic compounds exhibit AO properties (free radical scavenging activities) due to the presence of phenolic hydroxyl groups that are prone to donate a hydrogen atom or an electron to a free radical, and have an extended conjugated aromatic system to delocalize an unpaired electron [46]. Consequently, AO therapy is also a part of management protocols for T2DM and phenolics stands to be a much better aspirant [46–48]. In fact, phenolic compounds have been found to be more potent AOs than vitamin C, E and carotenoids *in vitro* [46]. Our study reports high TPC in BO and DC, and thus these TMPs may also possess high AO properties which supports its antidiabetic property. *BO* is noted to remain among the least investigated species in literature. However, some studies have reported BO to show highest phenolic content (2095 ± 120 mg of GAE/100 g) and thus highest 2,2-diphenyl-1-picrylhydrazyl (DPPH) radical scavenging activity compared with four other ferns investigated [49]. Phenolic compounds isolated from BO are mainly condensed tannins, terpenoids, flavonoid (Quercetin-7, 3′, 4′ trimethoxy) with potent AO properties [50–54]. While our study is the first to investigate the TPC of *DC*, work on TPC and AO activities on other *Dicranopteris* species, such as on *Dicranopteris linearis* has been reported [49,55]. Phenolics such as astragalin (main constituent), dichotomin A and B, kaempferol-3-*O*-(2″-*O*-*β*-*D*-glucopyranoside)-*β*-glucopyranoside and 4-vinyl phenol-1-*O*-(20-*O*-*α*-*L*-rhamnopyranosyl)-*β*-*D*-glucopyranoside has been isolated from this plant species [55]. Quercetin-7, 3′, 4′ trimethoxy present in these ferns may also contribute to its AO properties [56]. Our finding suggests that all ME extracts may have potential AOs and could be effective in managing T2DM associated complications resulting from ROS. To note, the present study was only limited to evaluating the TPC in the five TMPs and the AO property of these TMPs were not investigated [57,58]. However, these tests are currently under consideration.

### *In vitro* antidiabetic activity

**Figure 2:**
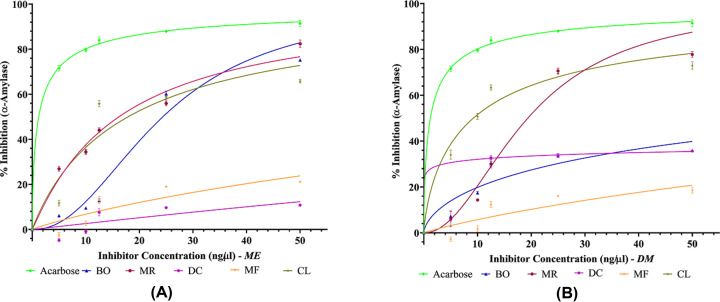
Inhibition of α-amylase activity by (A). ME plant extracts and (B). DM plant extracts of *Blechnum orientale* (BO), *Dicranopteris caudata* (DC), *Miscanthus floridulus* (MF), *Mussaenda raiateensis* (MR) and *Citrus limon* (CL) Acarbose (positive control) is used as reference drugs to compare the efficacy of the plant extracts. Concentration of plant samples taken: 5, 10, 12.5, 25 and 50 ng/µl. Results are expressed as mean ± SD (*n*=3); confidence interval = 95%. Graphs were plotted using nonlinear regression.

**Figure 3:**
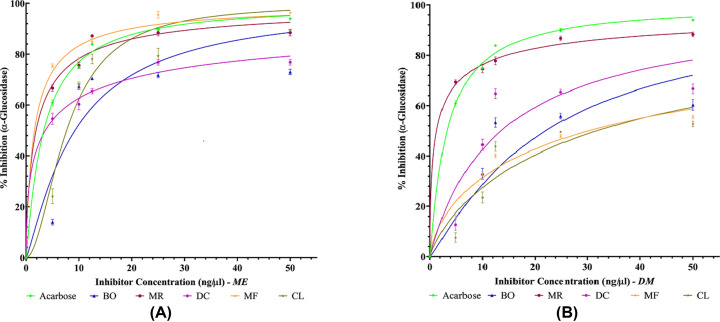
Inhibition of α-glucosidase activity by (A) ME plant extracts and (B) DM plant extracts of *Blechnum orientale* (BO), *Dicranopteris caudata* (DC), *Miscanthus floridulus* (MF), *Mussaenda raiateensis* (MR) and *Citrus limon* (CL) Acarbose (positive control) is used as reference drugs to compare the efficacy of the plant extracts. Concentration of plant samples taken: 5, 10, 12.5, 25 and 50 ng/µl. Results are expressed as mean ± SD (*n*=3), confidence interval = 95%. Graphs were plotted using nonlinear regression.

**Figure 4:**
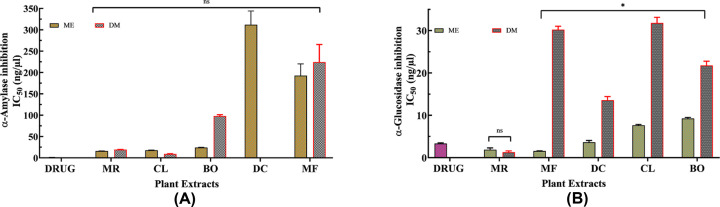
IC_50_ comparison of ME and DM plant extracts for (A) α-amylase inhibitory activities and (B) α-glucosidase inhibitory activities of *Blechnum orientale* (BO), *Dicranopteris caudata* (DC), *Miscanthus floridulus* (MF), *Mussaenda raiateensis* (MR) and *Citrus limon* (CL) with comparison to the drug (acarbose) Inversely proportional relationship between IC_50_ and enzyme activity is assessed. Statistical difference among the plants were assessed by one-way analysis of variance (ANOVA) and the individual comparisons were obtained by Turkey’s multiple comparisons test. Statistical significance was tested at a *P value < 0.05, for all cases in comparison with the positive control (acarbose); ns, not significant.

Dietary phenols, apart from their AO activity, have also been reported to exert antihyperglycemic effects by binding to glucose transporters [59] and competitively inhibiting digestive enzymes such as, α-amylase and α-glucosidase [21–23]. α-Amylase enzyme acts by cleaving the α-1,4 glycosidic linkage of polysaccharide (starch) to convert it to oligosaccharides such as maltose, which is further degraded by α-glucosidase enzyme to release absorbable monosaccharides such as glucose, fructose, and galactose. The monosaccharides are the only form of carbohydrate that can be absorbed into the blood in the small intestine [60,61]. Consequently, inhibiting these enzymes, would mean that there will be incomplete breakdown of the carbohydrate ingested in the meal. Hence, less monosaccharides will be released in the gut and so less monosaccharides absorbed. Thus, the longer PPHG that is characteristic of T2DM will be shorter and closer to normal [62]. Therefore, any source of inhibitors of these enzymes can delay carbohydrate digestion leading to reduced rate of glucose absorption and thus suppression of PPHG [63,64]. Acarbose is the most commonly prescribed enzyme inhibitory drug [13,14]. To compare the antidiabetic activity of the plants, α-amylase and α-glucosidase inhibitory test were performed. Comparison between percentage enzyme inhibitory activities of ME and DM plant extracts with acarbose is shown in Figures 2A,B and 3A,B, respectively. The percentage enzyme inhibitory activities displayed by acarbose and the five plants at concentration of 5, 10, 12.5, 25 and 50 ng/µl together with the IC_50_ (half-maximal inhibitory concentration) values is further shown in Supplementary Table S1a–d. IC_50_ value is the concentration of plant sample required for 50% enzyme inhibition. The IC_50_ comparative analysis between the plant extracts and acarbose for α-amylase and α-glucosidase is presented in Figure 4A,B.

All the MPs inactivated α-amylase and α-glucosidase enzyme in a dose dependant manner until the concentration of 25 ng/µl after which the inhibition activity neared to the saturation point. This is depicted in Figures 2A,B and 3A,B as the graph shows a plateau for majority of the MPs after 25 ng/µl. At highest plant concentration (50 ng/µl), none of the MPs were noted to be more potent than the standard drug, acarbose in inhibiting the activity of α-amylase. However, the MRME showed the highest α-amylase inhibitory activity reaching 82.44% inhibiting activity and IC_50_ = 16.18 ± 0.16 ng/µl. On the other hand, CLDM displayed the highest α-amylase inhibitory activity of 72.98% and IC_50_ = 9.21 ± 0.51 ng/µl. In comparison, ME of MF (96.22%, IC_50_ = 1.58 ± 0.03 ng/µl) and MR (88.63%, IC_50_ = 1.87 ± 0.43 ng/µl) inhibited α-glucosidase remarkably and much better than acarbose (93.92%, IC_50_ = 3.34 ± 0.15 ng/µl). Whilst MRDM *(*88.36%, IC_50_ = 1.31 ± 0.29 ng/µl) also showed outstanding α-glucosidase inhibitory activity. At 50 ng/µl, least α-amylase inhibitory activity was observed with DCME (10.79%) indicating that higher concentration of DC is required to achieve enzyme inhibitory activity, thus low potency in the specific antidiabetic property. However, all MPs under investigation showed remarkable α-glucosidase inhibitory activity with BO showing the least inhibition (73.02%) at 50 ng/µl. Few MPs showed the potential to significantly inhibit the enzyme activity at the lowest concentration of 5 ng/µl and compared well with the standard acarbose. At 5 ng/µl, the ME of DC, MF, MR inactivated α-glucosidase at 54.60%, 75.45% and 66.67% respectively. The MRDM inactivated α-glucosidase at 69.31%. Acarbose at 5 ng/µl inhibited the two enzymes, α-amylase and α-glucosidase, at 71.53% and 60.89% accordingly. It can be suggested that these MPs may possess high amount of enzyme inhibitors even at very low concentration of the crude plant extract. Due to crude extracts being used, this interesting result may be likely because of the phytochemicals working in a synergistic way to give the entire plant extract its therapeutic efficacy. Recent studies suggest that extract from entire MP has more benefits compared with the use of isolated compounds from the same MP extract [65,66].

In our study, 95% of MPs showed better α-glucosidase inhibition activity, when compared with α-amylase inhibition. In relation to MPs for T2DM, studies elsewhere have shown that MPs that have strong inhibition on α-glucosidase enzyme and less (or no) inhibition on α-amylase have better hypoglycemic therapy potential for direct use and/or to be developed into synthetic pharmacological drugs [67]. Thus, both MR and MF are potential candidates as they showed higher activity against the activity of α-glucosidase. This is consistent with a study reported on α-glucosidase activity displayed by *Rubus sanctus* [68]. Our study also noted that, MR may have both polar and less polar α-glucosidase inhibitory active compounds as both the extracts (ME and DM) displayed remarkable inhibitory activity.

While majority of the MPs investigated in the present study are novel, however, antidiabetic work on species of the same genus has been reported. For instance, leave extract of *Mussaenda roxburghii* showed remarkable α-glucosidase inhibitory activity [69] whereas root extract of *Mussaenda macrophylla* displayed antidiabetic and AO property [70]. Iridoids and triterpene saponins are common metabolites in *Mussaenda* species whereby quercetin, rutin, hyperin, ferulic acid, sinapic acid, β-sitosterol and saponin are found in *MR* [71,72]. Moreover, antidiabetic study on *Miscanthus sinensis* was noted to inhibit α-glucosidase [73]. The leaf and rhizome extracts of *Dicranopteris curranii* showed significant α-glucosidase inhibitory activity showing the presence of flavonoids, hydroxycinnamic acid and proanthocyanidin [53]. Moreover, rutin, gallic acid, methyl palmitate and shikimic acid from the leaves of *Dicranopteris linearis* acted synergistically producing a hepatoprotective effect, which also maybe contributed to this species anti-inflammatory and AO properties [55,74,75]. Our study reported α-amylase and α-glucosidase activity on roots of CL (IC_50_ = 7.65 ± 0.20 ng/µl). It is reported that essential oils from CL peels display better α-glucosidase inhibitory activity (IC_50_ = 7.56 µg/ml) than acarbose (IC_50_ = 8.44 µg/ml) when compared with its α-amylase inhibitory activity (IC_50_ = 8.16 µg/ml) [76]. Additionally, fruit of CL also showed a 100% α-glucosidase inhibitory activity whereby hesperidin was reported as the predominant phenolic compound [77]. Flavonoids such as quercetin, eriocitrin, didymin and naringin were further reported to be found in CL [78] whereby naringin and aglycone naringenin were reported as antidiabetic [79,80]. Antidiabetic study reported from literature on BO is largely limited. However, studies have isolated proanthocyanidin (class of polyphenols) to show strong radical scavenging (AO), antibacterial and anticancer properties [52]. The aqueous extract of the plant showed the presence of flavonoids, tannins and glycosides [81,82]. Moreover, topical application of BO was effective in treating diabetic ulcer wounds [51]. In one study where five selected edible and medicinal ferns were investigated for their α-glucosidase inhibitory activity, aqueous extract of BO showed a dose dependant enzyme inhibition together with highest content of proanthocyanidin [53]. The five plants investigated in our current study belongs to the same genus as the ones described above and reported in the literature. This strongly suggest that the plants may potentially have similar compounds and hence corroborating the observed optimal antidiabetic preference.

### Correlation analysis of TPC and antidiabetic activity

Plant phenols are effective in preventing T2DM via several pathways, apart from their AO potential [14,18,19,36]. Pearson correlation was applied to percentage crude yield, TPC, α-amylase and α-glucosidase activity as shown in Table 2.

**Table 2:** Pearson correlation coefficients was used to exhibit linear relationship among the total phenolic compounds, α-amylase and α-glucosidase activity in ME and DM extracts

Extract	α-Glucosidase	α-Amylase	TPC	α-Amylase (DM)	α-Glucosidase (DM)	% Crude yield (DM)
**ME**						
TPC	**0.384**	**0.414**				
% Crude yield			**−0.873**			**0.267**
α-Amylase	**- 0.447**			**0.861**		
α-Glucosidase					**0.401**	
**DM**						
TPC	**−0.223**	**0.861**				
% Crude Yield			**−0.594**			
α-Amylase	**−0.272**					

Interpretation of the size of a correlation coefficient [83] was done as follows: 1 ≥ *r* ≥ 0.9 (−1 to --0.9) indicates very strong positive (negative) correlation; 0.89 ≥ *r* ≥ 0.7 (−0.89 to --0.7) indicates strong positive (negative) correlation; 0.69 ≥ *r* ≥ 0.5 (−0.69 to --0.5) indicates moderate positive (negative) correlation; 0.49 ≥ *r* ≥ 0.3 (−0.49 to --0.3) indicates low positive (negative) correlation; 0.30 ≥ *r* ≥ 0.00 (−0.30 to 0.00) indicates negligible correlation.

Percentage crude yield of ME and DM plant extracts exhibited strong and moderate negative correlation with TPC, respectively (*r* = −0.873, *P*=0.05, *r* = −0.594, *P*=0.29; *n*=5). This suggests that high percentage yield of the ME or DM plant extracts do not necessarily mean high TPC of the crude plant extract. Thus, other secondary metabolites apart from phenols may have been extracted in both solvents that has led to the increased % crude yield of the crude extracts. Also, negligible correlation between the percentage crude yield of the ME and DM extracts was seen (*r* = 0.267, *P*=0.66, *n*=5). MPs may inhibit α-amylase and/or α-glucosidase; however, it has rarely been noted to display good inhibition for both α-amylase and α-glucosidase enzymes [84,85]. In the present study, a similar trend was seen. A moderately negative correlation was seen between the α-amylase and α-glucosidase activity of ME (*r* = −0.447, *P*=0.45, *n*=5) and negligible correlation between the α-amylase and α-glucosidase activity of DM plant extracts (*r* = −0.272, *P*=0.66, *n*=5). However, there was a moderately positive association between the α-glucosidase inhibitory activity in ME and DM (*r* = 0.401, *P*=0.5, *n*=5) and a strong positive correlation between α-amylase inhibitory activity in ME and DM (*r* = 0.861, *P*=0.06, *n*=5) plant extracts. Our study further associates TPC moderately positive with α-amylase and α-glucosidase (*r* = 0.414, *P*=0.49; *r*=0.384, *P*=0.52, *n*=5) in ME whereas, strongly positive with α-amylase (*r* = 0.861, *P*=0.06, *n*=5) in DM. Thus, our findings further indicate the presence of enzyme inhibitory compounds in both ME and DM extracts. On the other hand, TPC showed negligible correlation with α-glucosidase (*r* = −0.223, *P*=0.72, *n*=5) in DM. Other studies also reported a mixed outcome on correlation between TPC and inhibitory activity of the TMPs but does shows strong positive correlation between TPC and AO properties of TMPs [86,87]. Moreover, plant species with high phenolic content have also been reported to possess good AO capacity and high inhibitory activity against α-glucosidase enzyme [88].

## Conclusion

This is the first enzyme inhibition study on *Miscanthus floridulus, Mussaenda raiateensis and Dicranopteris caudate*. The present study provides the first pharmacological insight in the total phenolic content and antidiabetic potential of the selected Fijian traditional medicinal plants. Hydromethanolic extract of *Blechnum orientale* and *Dicranopteris caudate* showed highest total phenolic content compared with vanillic acid, thus displaying potential antioxidant capacity. *Mussaenda raiateensis* and *Miscanthus floridulus* inhibited α-glucosidase significantly compared to the most common drug, acarbose, indicating phytochemicals present in the extracts have potential to reduce postprandial hyperglycemia by delaying the carbohydrate digestion. The antioxidant capacity of these plants needs to be further explored using tests such as DPPH, ABTS and FRAP. Antidiabetic ability to inhibit α-amylase and α-glucosidase needs to be also further examined using *in vivo* experimental models for validation. Furthermore, discovery into the safety and effectiveness of the active antidiabetic compound needs to be made.

## Supplementary Material

Supplementary Table S1

## Data Availability

All data are available in the manuscript as well as Supplementary Material.

## Abbreviations

BO: *Blechnum orientale*
CL: *Citrus limon*
DC: *Dicranopteris caudata*
DM: Diabetes mellitus
DPPH: 2,2-diphenyl-1-picrylhydrazyl
MF: *Miscanthus floridulus*
MP: medicinal plant
MR: *Mussaenda raiateensis*
OS: oxidative stress
PPHG: postprandial hyperglycemia
ROS: reactive oxygen species
T1DM: Type 1 diabetes mellitus
T2DM: Type 2 diabetes mellitus
TPC: total phenolic content
