# Supplementary material for: Fijian medicinal plants and their role in the prevention of Type 2 diabetes mellitus
Source: Biosci Rep. 2022 Nov 16;42(11):BSR20220461. doi: 10.1042/BSR20220461 (PMC9670244; doi:10.1042/BSR20220461)
Supplement: Supplementary Table S1 [file BSR-2022-0461_supp.pdf]

SUPPLEMENTARY TABLE 1

Table 1a). Percentage α -amylase inhibition activity of ME.

Con (ng/µL)	*D.caudata*	*M.floridulus*	*M.raiateensis*	*C.limon*	*B.orientale*	Acarbose
5	-4.88±0.73	-2.35±0.98	26.88±1.08	11.68±1.31	6.16±0.28	71.53±1.20
10	-088±1.00	2.78±0.98	34.41±2.03	35.33±1.78	9.51±0.28	77.08±1.84
12.5	7.60±1.54	13.03±1.61	44.09±1.06	55.84±1.31	12.87±0.73	84.03±1.39
25	9.67±0.55	19.02±0.37	55.91±1.28	58.41±0.99	60.19±1.27	87.96±0.40
50	10.79±0.48	21.15±0.64	82.44±1.64	65.81±0.86	75.22±0.28	89.35±1.06
**IC_{50}**	**312.03±32.14**	**192.57±27.59**	**16.18 ± 0.16**	**17.89±0.52**	**24.38±0.39**	**1.21±0.23**

Table 1b). Percentage α -amylase inhibition activity of DM.

Con (ng/µL)	*D.caudata*	*M.floridulus*	*M.raiateensis*	*C.limon*	*B.orientale*	Acarbose
5	25.88±0.24	-2.78±0.98	6.09±3.46	34.09±2.00	7.31±2.12	71.53±1.20
10	28.41±0.24	1.71±1.33	14.34±0.62	50.76±1.31	17.44±0.65	77.08±1.84
12.5	32.77±0.97	12.39±1.33	30.11±1.86	63.38±1.16	30.10±1.06	84.03±1.39
25	33.47±0.65	16.03±0.59	70.61±1.24	69.44±0.44	33.76±0.73	87.96±0.40
50	35.72±0.24	18.59±1.28	77.78±1.24	72.98±1.58	35.87±0.16	89.35±1.06
**IC_{50}**	**ND***	**224.33±41.16**	**19.36 ± 0.43**	**9.21 ± 0.51**	**98.06±3.20**	**1.21 ± 0.23**

Table 1c). Percentage α -glucosidase inhibition activity of ME.

Con (ng/µL)	*D.caudata*	*M.floridulus*	*M.raiateensis*	*C.limon*	*B.orientale*	Acarbose
5	54.60±2.20	75.45±0.69	66.67±1.28	24.13±2.91	13.97±1.10	60.89±1.10
10	60.32±2.19	78.51±0.46	75.64±1.07	67.94±1.10	67.30±1.11	74.87±0.46
12.5	65.40±1.10	84.90±1.15	87.18±1.02	78.10±1.91	70.48±0.13	83.92±0.49
25	76.83±1.12	95.47±1.33	88.46±1.28	79.37±2.91	71.75±1.17	89.95±0.61
50	76.83±1.10	96.22±0.58	88.63±1.39	88.25±1.10	73.02±1.10	93.92±0.46
**IC_{50}**	**3.63 ± 0.42**	**1.58 ± 0.03**	**1.87 ± 0.43**	**7.65 ± 0.20**	**9.25 ± 0.25**	**3.34 ± 0.15**

Table 1d). Percentage α -glucosidase inhibition activity of DM.

Con (ng/µL)	*D.caudata*	*M.floridulus*	*M.raiateensis*	*C.limon*	*B.orientale*	Acarbose
5	12.70±2.20	15.81±0.74	69.31±0.92	7.62±1.91	-10.80±1.10	60.89±1.10
10	44.442±2.19	32.91±0.45	74.60±1.59	23.49±2.20	33.02±2.20	74.87±0.46
12.5	64.76±1.91	40.17±0.75	77.78±1.59	43.81±1.91	53.33±1.91	83.92±0.49
25	65.40±1.10	47.86±0.61	86.77±0.92	49.52±0.61	55.87±1.10	89.95±0.61
50	66.67±1.91	55.56±0.74	88.36±0.23	52.70±1.10	60.32±2.20	93.92±0.46
**IC_{50}**	**13.60 ± 0.82**	**30.24 ± 0.79**	**1.31 ± 0.29**	**31.80±1.33**	**21.78±1.00**	**3.34 ± 0.15**
